# Latino adolescent-father discrepancies in reporting activity parenting practices and associations with adolescents’ physical activity and screen time

**DOI:** 10.1186/s12889-020-8199-6

**Published:** 2020-01-21

**Authors:** Youjie Zhang, Aysegul Baltaci, Francine Overcash, Stephanie Druziako, Alejandro Peralta, Ghaffar Ali Hurtado, Marla Reicks

**Affiliations:** 1 Soochow University Medical College, Suzhou, China; 2 University of Minnesota Twin Cities, St. Paul, USA; 3 University of Maryland College Park, College Park, USA

## Abstract

**Background:**

Latino fathers may play important roles in adolescents’ physical activity and screen time. However, informant discrepancies regarding paternal activity parenting practices may challenge studies supporting evidence-based applications. This study examined Latino adolescent-father discrepancies in reporting paternal activity parenting practices, types of discrepancies by participant characteristics, and associations between discrepancy types and adolescents’ physical activity and screen time.

**Methods:**

The sample for this cross-sectional study included Latino early adolescents and their fathers (*n* = 138 dyads) from baseline data collected for a family-centered, healthy lifestyle intervention in a metropolitan area. In parallel measures, Latino adolescents and fathers reported paternal activity parenting practices related to expectation or allowance, behavioral modeling, and providing opportunities for physical activity or screen time. Level of agreement and discrepancies were examined using the percentage of agreement, weighted kappa statistics, Pearson correlation coefficients, and paired-sample t-tests. Undesirable discrepancy types included adolescents reporting lower scores for paternal physical activity parenting practices or higher scores for paternal screen time parenting practices than fathers. Participants’ sociodemographic characteristics and weight status were compared by discrepancy type using between-group t-tests or Chi-square tests. Associations between discrepancy type and adolescents’ physical activity and screen time were examined using multivariate regression analyses.

**Results:**

The study sample was low-income with a high prevalence of overweight and obesity. Adolescent and paternal reports of activity parenting practices had poor agreement (percentages of agreement: 22.2–34.3%, weighted kappa statistics: < 0.2, and correlation coefficients: 0.06–0.25). An undesirable discrepancy type for certain parenting practices was more likely to be observed among fathers without full-time employment, girls, older adolescents, and adolescents and fathers within overweight or obese BMI categories. Discrepancies in paternal expectation regarding physical activity and allowance of screen time had adverse associations with adolescents’ physical activity (β = − 0.18, *p* = 0.008) and screen time (β = 0.51, *p* <  0.001).

**Conclusion and implications:**

Discrepancies in reporting activity parenting practices were evident between Latino adolescents and their fathers, especially among certain sociodemographic and weight status groups. Adolescents’ perceptions on paternal parenting practices tended to be better indicators of their activity levels than fathers’ reports.

## Introduction

Parents are important stakeholders for fostering healthy lifestyle behaviors among children and early adolescents. Various parenting practices have been used to manage children and adolescents’ physical activity (PA) and screen time (ST) [1, 2]. Common activity parenting practices included setting rules or limits, modeling, and practices related to availability and accessibility [2, 3]. However, these parenting practices demonstrated only modest or inconsistent associations with children and adolescents’ PA and ST in survey studies [2, 4, 5]. Researchers have expressed concerns about measurement issues related to activity parenting practices such as lack of consensus on construct conceptualization and adequate reliability and validity of construct operationalization, which may hamper the assessment of parental influences [4, 6].

Another concern about the measurement of activity parenting practices is informant discrepancy, which occurs when multiple informants provide inconsistent responses to assessment of the same behavior [7]. Indeed, several studies with adolescent-parent dyads have found poor agreement in reports of familial support for physical activity and limitations on screen time between adolescents and their parents [8–10]. However, prior studies of activity parenting practices were primarily based on either adolescent or parent reporting [4, 11], without considering the potential influence of informant discrepancy.

Informant discrepancy can lead to different conclusions and challenge empirical application of evidence-based practices [12]. Discrepancies in parent and adolescent responses have a complex nature beyond measurement error based on potential social desirability bias [13]. Informant discrepancy may also reflect adolescents’ natural development toward autonomy and independence, and problems in adolescent-parent relationships such as lack of effective communication and responsive parenting [14]. Therefore, examination of informant discrepancy regarding activity parenting practices is necessary to effectively involve parents in promoting active lifestyles among children and adolescents.

Latino adolescents are vulnerable to unhealthy weight gain in the United States [15], which may be partially attributed to a relatively sedentary lifestyle. Accelerometer data showed that a representative sample of Latino youth aged 8–16 in the United States, on average, had 35 min of moderate to vigorous physical activity (MVPA) per day, which was below the recommendation of 60 min [16]. In addition, more than three-quarters of Latino adolescents aged 12–19 in the United States had more than two hours of daily screen time [17].

Latino fathers may play an important role in influencing early adolescents’ physical activity and screen time. Qualitative studies found that Latino fathers valued the health and developmental benefits of physical activity and applied a variety of practices to engage in physical activity with their children and limit screen time [18–20]. However, Latino adolescents’ perceptions of their fathers’ parenting practices may differ from their fathers’ perceptions. Two previous studies found poor agreement in reporting family functioning between Latino adolescents and their parents [21, 22]. These studies found that negative discrepancies between adolescents and parents in reporting family functioning were associated with adverse youth behavioral outcomes, such as less physical activity [21] and more delinquent behaviors [22]. However, Latino adolescents’ perceptions regarding paternal activity parenting practices remain unknown. Quantitative data that assess Latino paternal activity parenting practices and discrepancies between adolescents’ perceptions and paternal reports, as well as an examination of the potential influence on adolescents’ activity levels are lacking.

Therefore, the present study aimed to 1) identify informant discrepancies in reporting paternal activity parenting practices between Latino early adolescents and their fathers, 2) identify socio-demographic and weight-related correlates of adolescent-father discrepancies, and 3) examine associations between the type of discrepancy (categorized by positive, negative or matching scores for adolescents’ and fathers’ reports of paternal activity parenting practices) with adolescents’ physical activity and screen time.

## Method

### Participants

This cross-sectional study included a sample of 138 Latino adolescent-father dyads from the baseline data collection of the “Padres Preparados, Jóvenes Saludables” program, a community-based, family-centered, healthy lifestyle intervention [23]. Participants were recruited using flyers and announcements at community service centers and churches in the Minneapolis/St. Paul, Minnesota metropolitan area between September 2017 and March 2019. Eligibility criteria included adolescents between 9 and 14 years of age, and fathers 1) having meals with the adolescent at least three times in a week, 2) self-identifying as Latino, and 3) speaking Spanish. These eligibility criteria were consistent for the intervention program as well as for the current study. The criterion of “having meals with the adolescent at least three times in a week” was applied to ensure that fathers were present in the home to interact with the adolescent for some period of time given the tendency for fathers to work multiple jobs and have irregular schedules. Consent and assent were obtained prior to data collection, and adolescents and fathers received 25 USD and 35 USD, respectively, as compensation for their participation. The study was approved by the University of Minnesota Institutional Review Board Human Subjects Protection Committee.

### Paternal activity parenting practices

**Fig. 1:**
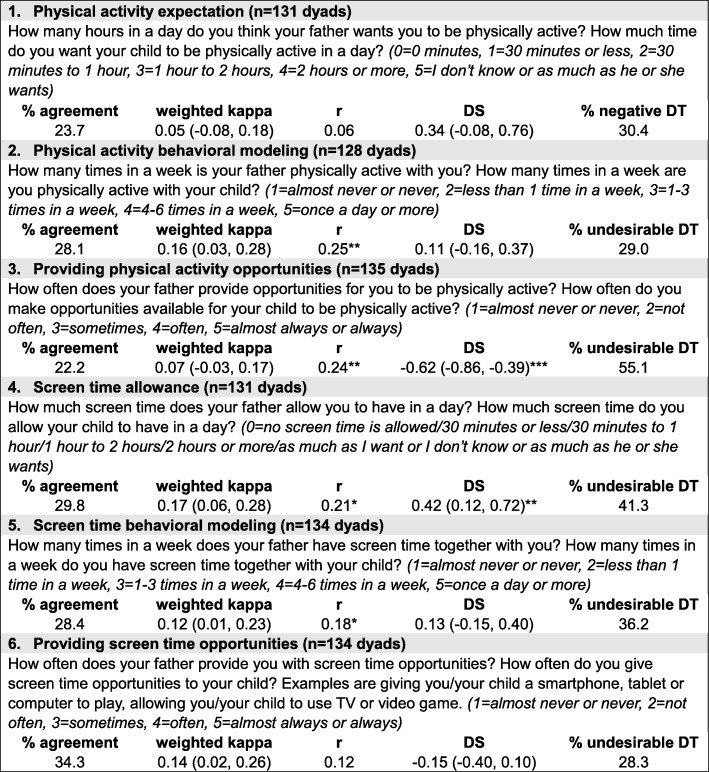
Measures of activity parenting practices from adolescent and father surveys and agreements and discrepancies between adolescent and paternal reports (r: Pearson correlation coefficients; DS: discrepancy scores calculated using paternal scores subtracted from adolescent scores; undesirable DT: a undesirable discrepancy type refers to adolescents’ scores lower than fathers’ scores in reporting physical activity parenting practices and adolescents’ scores higher than fathers’ scores in reporting screen time parenting practices. * *p* < 0.05, **p < 0.01, *** *p* < 0.001)

Adolescents’ perceptions of paternal parenting practices around their physical activity were assessed using items related to setting an expectation, behavioral modeling, and providing opportunities for physical activity (Fig. 1). A similar set of items was used to assess adolescents’ perceptions of paternal parenting practices involving screen time: allowing ST, behavioral modeling and providing opportunities for ST. These survey items were developed based on existing instruments and focus group findings, and demonstrated acceptable criterion validity against adolescents’ MVPA and ST [24]. Parallel questions were designed to assess paternal reports of these parenting practices. Strategies to reduce response bias included cognitive testing among four adolescents and five fathers, revising survey questions to facilitate comprehension, informing participants that there were no right or wrong answers, and having adolescents and fathers complete the surveys in separate locations to minimize influence from family members.

### Adolescents’ physical activity

Adolescents self-reported their physical activity levels in response to three survey questions assessing hours spent in a usual week on vigorous, moderate, and mild exercises with examples provided for each level [25, 26]. Response options (0, < 30 min, 0.5 to 2 h, 2.5 to 4 h, 4.5 to 6 h, and > 6 h) were recoded to 0, 0.3, 1.3, 3.3, 5.3, and 8, respectively. Total daily physical activity hours were determined by summing the three items and then dividing by seven days. Total physical activity hours for adolescents (*n* = 4) who reported > 2 h daily were top coded to 2 h. This cut-off point was located at the 99th percentile of the distribution. Test-retest correlations of these items ranged from of 0.51 to 0.69 [26].

### Adolescents’ screen time

Adolescents self-reported screen time in hours spent watching TV/DVDs/videos; using a computer (not for homework or work), playing electronic games while sitting, and using smartphones and tablets on a typical weekday and weekend day [27]. Seven response categories ranged from 0 h to > 5 h for each type of screen time. Total daily screen time hours were calculated using a weighted sum of weekday and weekend day screen time hours divided by seven days. Test-retest correlations of these items ranged from of 0.60 to 0.80 [27]. Screen time hours for adolescents (*n* = 17) who reported > 10 h of recreational screen time per day were top coded to 10 h. This cut-off point was determined by examining the distribution of participants’ responses as well as consideration of responses indicating implausible reporting and/or multi-tasking.

### Sociodemographic variables

Sociodemographic variables included adolescents’ age and sex; and fathers’ education, employment status, length of stay in the United States, language spoken at home, household annual income, and neighborhood safety. An acculturation score ranging from 0 to 5 was created based on father-reported length of stay in the U.S. and language spoken at home [28]. Low and high acculturation status were defined as scores ranging from 0 to 2 and from 3 to 5, respectively. Neighborhood safety was determined using two items adapted from the Neighborhood Environment Walkability Scale [29]. A mean score > 2 indicated an unsafe neighborhood for walking and ≤ 2 indicated a safe neighborhood.

### Anthropometric measures

Trained research assistants measured adolescents’ and fathers’ weight and height according to standardized methods used for the U.S. National Health and Nutrition Examination Survey [30]. Adolescents’ sex- and age-specific Body Mass Index (BMI) percentiles and categories were determined according to the 2000 CDC growth charts [30]. Fathers’ BMI was calculated using weight (kg) divided by height in meters squared (m^2^) and categorized into three groups: < 25 kg/m^2^ (under or normal weight), 25 to 29.9 kg/m^2^ (overweight) and ≥ 30 kg/m^2^ (obese) [31].

### Statistical analysis

Statistical analysis was performed using SAS 9.4 (Cary, NC, USA, 2002–2012). Descriptive analyses were performed on sociodemographic variables, adolescents’ weight status, scores of paternal parenting practices and adolescents’ physical activity and screen time. Square root transformations were performed on adolescents’ daily MVPA hours and screen time hours to improve the normality of the distribution.

Agreements between adolescent and paternal reports of activity parenting practices were assessed using percentages of exact match, weighted kappa statistics, Pearson correlation coefficients, and paired-group t-tests. Discrepancy scores were calculated by subtracting father’s scores from adolescent’s scores for each activity parenting practice. Then, the type of discrepancy was determined by discrepancy scores ≥0 (matched or positive) and <  0 (negative or an undesirable type) for physical activity parenting practices; and ≤ 0 (matched or negative) and >  0 (positive or an undesirable type) for screen time parenting practices. The scoring system was based on previous findings which showed that Latino adolescents with worse perceptions about parenting than their parents had less desirable behavioral outcomes [21, 22]. Sociodemographic characteristics and adolescents’ and fathers’ BMI categories were compared by discrepancy type for each parenting practice using between-group t-tests and chi-square tests. Lastly, multivariate regression analyses were performed to examine associations between discrepancy type and adolescents’ physical activity and screen time. Covariates were adolescents’ age (≤ 12 vs. > 12 years old) and sex (boy vs. girl), fathers’ education (< high school vs. ≥ high school), employment status (full-time or not) and acculturation level (high vs. low), household annual income (< $35,000 vs. ≥ $35,000) and neighborhood safety (safe vs. unsafe), and adolescents’ and fathers’ BMI categories (overweight/obese or not). These covariates had significant relationships with at least one discrepancy type or adolescents’ physical activity or screen time. Instead of excluding dyads with any missing data at one time, each statistical analysis addressed missing values separately. Post hoc statistical power was calculated using G*Power 3.1 [32]. The current study reached a power of at least 92% for paired group comparisons of means with an effect size of 0.3 and a power of at least 91% for multivariate regression with an effect size of 0.1 and 9 predictors. Significance levels were set at *p* <  0.05, two-tailed.

## Results

### Participant characteristics

In the sample of 138 Latino adolescent-father dyads, adolescents had a mean age of 12 years (all were 9–14 years except for three who were 15 or 16 years). Adolescents were approximately equally distributed by sex (Table 1). Fathers had a mean age of 41 and most had a high school education or less (80%). Over 85% of fathers reported an annual household income less than $50,000, and about 68% of fathers reported having full-time employment. Most fathers had low acculturation status and primarily or only spoke Spanish or their native language at home. Nearly a third of adolescents had BMI percentiles above 85% and nearly 90% of fathers had a BMI 25 kg/m^2^ or higher. In addition, only small fractions of adolescents reported ≥1 h of MVPA (*n* = 19, 13.8%) and ≤ 2 h of screen time (*n* = 31, 22.5%) on an average day.

**Table 1:** Participants’ sociodemographic characteristics and weight status and adolescents’ daily physical activity (PA) hours, and screen time hours (*n* = 138)

Characteristics	Count (%^a^) or mean ± SD
Adolescent boys	72 (52.2)
Adolescents’ age	11.6 ± 1.5
Fathers’ age	41.4 ± 7.2
Fathers’ marital status	
Married	114 (82.6)
Living with a partner	9 (6.5)
Single or divorced	11 (8.7)
Fathers’ education	
Lower than high school	53 (38.4)
High school, GED	59 (42.8)
Some college or higher	24 (17.4)
Annual household income	
< $24,999	55 (39.9)
$25,000 to $49,999	59 (42.8)
$50,000 to $99,999	18 (13.0)
Employment status	
Self-employed	23 (16.7)
Unemployed, homemaker	6 (4.4)
Part-time	11 (8.0)
Full-time	94 (68.1)
Years in U.S.	19.1 ± 5.4
< 10 years	2 (1.5)
10 to < 20 years	79 (57.3)
20 to < 30 years	48 (34.8)
≥ 30 years	6 (4.4)
Language spoken at home	
Primarily or only native language	110 (79.7)
Equally native language and English	23 (16.7)
Primarily or only English	3 (2.2)
Acculturation (range: 0–5)	1.6 ± 0.7
Low	119 (86.2)
High	16 (11.6)
Neighborhood Safety	
Unsafe	56 (40.6)
Safe	73 (52.9)
Adolescents’ BMI categories	
< 85th percentile	54 (39.1)
85th – 94.9th percentile	31 (22.5)
≥ 95th percentile	49 (35.5)
Fathers’ BMI categories	
< 25 kg/m^2^	14 (10.1)
25–29.9 kg/m^2^	61 (44.2)
≥ 30 kg/m^2^	59 (42.8)
Behaviors	Median (IQR) or count (%)
Adolescents’ daily PA hours	0.23 (0.09, 0.51)
Having ≥ 1 h/day of PA	19 (13.8)
Adolescents’ daily screen time hours	3.93 (2.21, 7.57)
Having ≤2 h/day of screen time	31 (22.5)

^a^percentages may not add up to 100% due to missing values

### Agreements and discrepancies in activity parenting practices between adolescents and fathers

Adolescent and paternal reports of activity parenting practices showed poor agreement as indicated by low percentages of exact matches (22.2–34.3%), weighted kappa statistics (< 0.2) and weak correlation coefficients (0.06–0.25, Fig. 1). However, the discrepancy scores did not show signs of directionality except that adolescents reported significantly lower frequencies of fathers providing physical activity opportunities and higher amounts of time that fathers allowed screen time than their fathers did (− 0.62, *p* <  0.0001 and 0.42, *p* <  0.01, respectively). About 30 to 50% of the dyads were described as having the undesirable type of discrepancy in reporting.

### Participants’ sociodemographic characteristics and weight status by discrepancy types for paternal activity parenting practices

The proportion of dyads having undesirable discrepancies in scores for behavioral modeling of PA was higher among fathers without full-time employment than those with full-time employment (48.7% vs. 24.7%, *p* <  0.01) (Table 2). No other sociodemographic characteristics or weight status differed by discrepancy type, however the proportion of dyads having undesirable discrepancy scores for paternal expectations for physical activity tended to be higher among adolescents with a BMI ≥ the 85th percentile than those with a BMI < the 85th percentile (37.3% vs. 23.1%, *p* = 0.09). The proportion of dyads exhibiting undesirable discrepancy scores for providing physical activity opportunities also tended to be higher among families who lived in a safe neighborhood compared to those who lived in an unsafe neighborhood (62.0% vs. 46.4%, *p* = 0.08).

**Table 2:** Participants’ sociodemographic characteristics and weight status by discrepancy types for paternal parenting practices related to adolescents’ physical activity (PA), *n* = 138^a^

Characteristics	Discrepancy type by discrepancy scores^b^								
	Expectation		*p*-value	Behavioral modeling		*p*-value	Providing opportunity		*p*-value
	< 0	≥ 0		< 0	≥ 0		< 0	≥ 0	
Adolescents’ sex									
Boy	21 (29.6)	50 (70.4)	0.55	18 (26.1)	51 (73.9)	0.27	41 (56.9)	31 (43.1)	0.74
Girl	20 (34.5)	38 (65.5)		20 (35.1)	37 (64.9)		33 (54.1)	28 (45.9)	
Adolescents’ age	11.3 ± 1.5	11.7 ± 1.6	0.19	11.6 ± 1.6	11.5 ± 1.5	0.59	11.4 ± 1.4	11.7 ± 1.6	0.31
Fathers’ age	42.0 ± 6.8	41.1 ± 7.4	0.47	42.2 ± 8.2	41.3 ± 6.9	0.55	40.9 ± 7.0	42.1 ± 7.5	0.33
Fathers’ education									
Middle school or lower	18 (36.0)	32 (64.0)	0.39	19 (38.8)	30 (61.2)	0.16	29 (55.8)	23 (44.2)	0.97
High school or higher	23 (28.8)	57 (71.3)		21 (26.9)	57 (73.1)		46 (56.1)	36 (43.9)	
Family income									
< $35,000	25 (32.1)	53 (68.0)	0.88	22 (29.3)	53 (70.7)	0.48	44 (53.7)	38 (46.3)	0.33
≥ $35,000	16 (33.3)	32 (66.7)		17 (35.4)	31 (64.6)		30 (62.5)	18 (37.5)	
Fathers’ employment									
Not full-time	12 (30.0)	28 (70.0)	0.77	18 (48.7)	19 (51.3)	**< 0.01**	25 (62.5)	15 (37.5)	0.27
Full-time	29 (32.6)	60 (67.4)		22 (24.7)	67 (75.3)		48 (52.2)	44 (47.8)	
Fathers’ acculturation score	1.6 ± 0.7	1.6 ± 0.8	0.97	1.7 ± 0.7	1.6 ± 0.8	0.81	1.7 ± 0.7	1.7 ± 0.8	0.99
Neighborhood safety									
Unsafe	14 (25.9)	40 (74.1)	0.27	14 (26.9)	38 (73.1)	0.36	26 (46.4)	30 (53.6)	0.08
Safe	25 (35.2)	46 (64.8)		24 (34.8)	45 (65.2)		44 (62.0)	27 (38.0)	
Adolescents’ BMI categories									
< 85%th	12 (23.1)	40 (76.9)	0.09	16 (32.7)	33 (67.4)	0.70	33 (62.3)	20 (37.7)	0.27
≥ 85%th	28 (37.3)	47 (62.7)		22 (29.3)	53 (70.7)		41 (52.6)	37 (47.4)	
Fathers’ BMI categories									
< 30 kg/m^2^	23 (32.9)	47 (67.1)	0.83	25 (36.2)	44 (63.7)	0.18	41 (55.4)	33 (44.6)	0.71
≥ 30 kg/m^2^	18 (31.0)	40 (69.0)		14 (25.0)	42 (75.0)		34 (58.6)	24 (41.4)	

Statistical comparisons were conducted using between-group t-tests and chi-square tests

Significant *p*-values are in bold

^a^Sample size of each statistical comparison varied due to missing values

^b^Undesirable discrepancy type for physical activity = discrepancy scores < 0

The proportion of dyads having undesirable discrepancy scores for allowance of screen time was higher among fathers without full-time employment than those with full-time employment (64.1% vs. 35.2%, *p* = 0.003, Table 3). A similar tendency was shown by adolescents’ sex (51.7% for girls vs. 35.2% for boys, *p* = 0.06). In addition, adolescents with undesirable discrepancy scores were older (12.1 vs. 11.1, *p* < 0.001). Older adolescents were also more likely to have undesirable discrepancy scores for paternal behavioral modeling than younger adolescents (11.8 vs. 11.3, *p* < 0.05). The proportion of dyads with undesirable discrepancy scores for providing screen time opportunities was higher among adolescents with a BMI ≥ the 85th percentile than those with a BMI < the 85th percentile (35.4% vs. 15.7%, *p* = 0.01) as well as among fathers with a BMI ≥ 30 kg/m^2^ than fathers with a BMI < 30 kg/m^2^ (40.7% vs. 18.1%, *p* = 0.004). In addition, there was a tendency for adolescents with undesirable discrepancy scores to be younger (11.2 vs. 11.7 *p* = 0.06).

**Table 3:** Participants’ sociodemographic characteristics and weight status by discrepancy types for paternal parenting practices related to adolescents’ screen time (ST), *n* = 138^a^

Characteristics	Discrepancy type by discrepancy scores^b^								
	Allowance		*p*-value	Behavioral modeling		*p*-value	Providing opportunity		*p*-value
	> 0	≤ 0		> 0	≤ 0		> 0	≤ 0	
Adolescents’ sex									
Boy	25 (35.2)	46 (64.8)	0.06	29 (40.9)	42 (59.2)	0.34	22 (31.0)	49 (69.0)	0.41
Girl	30 (51.7)	28 (48.3)		20 (32.8)	41 (67.2)		15 (25.0)	46 (75.0)	
Adolescents’ age	12.1 ± 1.4	11.1 ± 1.6	**< 0.001**	11.8 ± 1.6	11.3 ± 1.5	**< 0.05**	11.2 ± 1.4	11.7 ± 1.6	0.07
Fathers’ age	42.1 ± 7.2	40.9 ± 7.3	0.34	42.2 ± 7.1	41.1 ± 7.3	0.37	42.4 ± 6.2	41.0 ± 7.6	0.28
Fathers’ education									
Middle school or lower	22 (44.9)	27 (55.1)	0.85	21 (40.4)	31 (59.6)	0.59	16 (31.0)	36 (69.2)	0.77
High school or higher	35 (43.2)	46 (56.8)		29 (35.8)	52 (64.2)		23 (28.4)	58 (71.6)	
Family income									
< $35,000	34 (42.5)	46 (57.5)	0.73	34 (42.0)	47 (58.0)	0.33	28 (33.7)	55 (66.3)	0.15
≥ $35,000	21 (45.7)	25 (54.4)		16 (33.3)	32 (66.7)		10 (21.7)	36 (78.3)	
Fathers’ employment									
Not full-time	25 (64.1)	14 (35.9)	**0.002**	16 (41.0)	23 (59.0)	0.58	14 (35.9)	25 (64.1)	0.32
Full-time	31 (34.4)	59 (65.6)		33 (35.9)	59 (64.1)		25 (27.2)	6 (72.8)	
Fathers’ acculturation score	1.7 ± 0.7	1.5 ± 0.8	0.15	1.8 ± 0.7	1.6 ± 0.8	0.13	1.7 ± 0.7	1.6 ± 0.8	0.63
Neighborhood safety									
Unsafe	27 (50.9)	26 (49.1)	0.15	18 (33.3)	36 (66.7)	0.52	17 (30.4)	39 (69.6)	0.66
Safe	27 (38.0)	44 (62.0)		28 (38.9)	44 (61.1)		19 (26.8)	52 (73.2)	
Adolescents’ BMI categories									
< 85%th	20 (40.0)	30 (60.0)	0.54	20 (39.2)	31 (60.8)	0.66	8 (15.7)	43 (84.3)	**0.01**
≥ 85%th	35 (45.5)	42 (54.6)		28 (35.4)	51 (64.6)		28 (35.4)	51 (64.6)	
Fathers’ BMI categories									
< 30 kg/m^2^	34 (48.6)	36 (51.4)	0.14	26 (35.6)	47 (64.4)	0.50	13 (18.1)	59 (81.9)	**0.004**
≥ 30 kg/m^2^	21 (35.6)	38 (64.4)		24 (41.4)	34 (58.6)		24 (40.7)	35 (59.3)	

Statistical comparisons were conducted using between-group t-tests and chi-square tests

Significant *p*-values are in bold

^a^Sample size of each statistical comparison varied due to missing values

^b^Undesirable discrepancy type for screen time = discrepancy scores > 0

### Association between discrepancy types for activity parenting practices and adolescents’ physical activity and screen time

Multivariate regression models showed that the undesirable type of informant discrepancy for paternal expectation regarding physical activity was inversely associated with adolescents’ daily MVPA (β = − 0.18, *p* = 0.008), while discrepancy type for paternal behavioral modeling and providing opportunities did not show significant associations with adolescents’ daily MVPA. Similarly, the undesirable discrepancy type for paternal allowance of screen time was associated with greater adolescents’ daily screen time (β = 0.51, *p* < 0.001), but discrepancy type for the other paternal screen time parenting practices did not show significant associations with adolescents’ daily screen time.

## Discussion

The present study examined informant discrepancies in reporting activity parenting practices among a sample of adolescent-father dyads from Latino immigrant families with low socioeconomic status and high rates of overweight and obesity. In general, adolescents and their fathers disagreed in their reports of paternal activity practices. The discrepancy type was significant by several sociodemographic characteristics and weight status. The type of discrepancy for paternal expectation regarding physical activity and allowance of screen time was associated with adolescent-reported MVPA and screen time, respectively. Findings from this study expanded current knowledge of informant discrepancy regarding perceptions of paternal activity parenting practices related to adolescents’ physical activity and screen time with a focus on Latino fathers and early adolescents.

Agreement between Latino early adolescents and fathers on reporting activity parenting practices were generally poor regardless of the type of activity or parenting practice. One previous study conducted among black adolescent girls and their parents examined familial support for physical activity using ten survey items that combined parenting constructs of setting expectations, behavioral modeling and providing opportunities [9]. This study found poor adolescent/parent agreement with weighted kappa statistics lower than 0.2. However, the study used unmatched measures to compare adolescent perceptions about supports for physical activity from their families with reports of supports provided by one of their parents [9]. The present study addressed this limitation by designing parallel items for each parenting construct and ensuring content validity through cognitive testing [24]. Nevertheless, informant discrepancy still persisted in the current study. In fact, informant discrepancy is common in the field of family studies [7]. Meta-analyses with synthesized correlations and difference scores demonstrated poor agreement between parent- and child-reported general parenting constructs [13, 33]. Therefore, the present study demonstrated that informant discrepancy between adolescents and parents might also be common regarding paternal activity-related parenting practices.

From a methodological perspective, parents are likely under the influence of social desirability and report their parenting practices in a manner that would support better adolescent behavior outcomes [33]. For example, the study by Wang and colleagues found that parents reported significantly higher familial support for physical activity than their black adolescent girls did [9]. However, this pattern of discrepancy was not found in most of the activity parenting practices examined in the present study with Latino early adolescents and fathers, except for providing physical activity opportunities and allowing screen time. This phenomenon indicated that discrepancy in reporting paternal activity parenting practices might be complicated by various factors in addition to social desirability. These factors could include adolescents’ cognitive ability in understanding parents’ perspectives, adolescent-parent relationships and the lack of effective communication [14]. How these factors influence adolescent-parent congruency in reporting parenting practices deserves further investigation and may shed light on effective strategies for improving paternal activity parenting practices.

Adolescent-father dyads were grouped by two types of discrepancy scores based on previous findings showing that Latino adolescents with worse perceptions about parenting than their parents showed less desirable behavioral outcomes [21, 22]. In the current study, undesirable discrepancy types for each activity parenting practice was prevalent for about one-third to one-half of the sample. Some of the undesirable discrepancy types were more prevalent in certain sociodemographic and weight status groups. For example, more girls and older adolescents had undesirable discrepancy scores for fathers allowing screen time than boys and younger adolescents. One study found that Mexican American fathers monitored fifth graders’ activities to a lesser extent for girls than boys [34]. This may account for Latino girls in the current study being more likely to perceive that their fathers were more permissive about restricting screen time than boys. Or girls may generally have less screen time than boys and are less engaged in activities such as computer games which parents may feel the need to control [35]. The difference by age could be related to parents exerting fewer prohibitory behaviors in response to adolescents’ developmental needs for greater autonomy and independence [36]. In addition, more fathers without full-time employment had undesirable discrepancy scores for physical activity behavioral modeling and allowing screen time than fathers with full-time employment. This may indicate that the stress from an unstable employment status could have affected these paternal parenting practices without the fathers’ awareness. Moreover, higher proportions of adolescents and fathers with overweight or obese weight status had undesirable discrepancy scores for providing screen time opportunities. This may reflect a greater perception by adolescents that fathers were providing screen time availability when the adolescents or their fathers were in unhealthy BMI categories. Variations in the distribution of discrepancy types by participant sociodemographic characteristics and weight status may provide direction for enhancing paternal activity parenting practices with emphases on certain groups.

General parenting studies suggested that adolescent-parent discrepancies may influence adolescents’ behavioral outcomes [13]. When adolescents had worse perceptions than parents, it was considered a manifestation of poor familial functioning [13, 21]. In the field of childhood obesity research, familial functioning has shown protective effects on adolescents’ weight status, dietary behaviors and physical activity [37, 38]. Likewise, discrepancies in reporting family functioning were associated with reduced physical activity and fruit and vegetable intake among adolescents [21]. Beyond general parenting, the present study found that discrepancies in paternal expectation for physical activity and allowing screen time showed similar adverse associations with adolescents’ physical activity and screen time, respectively. However, discrepancy types for other paternal activity parenting practices did not show this relationship. This difference may indicate paternal expectation for physical activity and rules about screen time may be more influential than behavioral modeling and providing opportunities for adolescents who are at the development stage of increasing autonomy and independence. Further investigation may be useful to determine whether improvements regarding discrepancies in adolescent-father reports of these paternal activity parenting practices would have positive effects on adolescents’ behavioral outcomes. If fathers have expectations that their adolescents are active and limit their screen time, and adolescents have an understanding consistent with these expectations, they may be encouraged to be lead active lifestyles.

This study has several limitations. Adolescents’ report of activity levels is likely to be in the same direction as their reported parenting practices. A lack of objective assessment of adolescents’ activity levels can introduce response bias related to this same informant effect, even though one study found that adolescents’ MVPA measured by accelerometers was associated with familial support for physical activity reported by adolescents but not by parents [9]. In addition, a cross-sectional design cannot infer causal relationships from associations between discrepancy types and adolescents’ behaviors. This design also limited further explanation of the significant age effects on the discrepancies for reporting screen time parenting practices. Future studies need to apply a longitudinal design to investigate whether adolescent-father discrepancies progress with age. A better resolution of informant discrepancy could be combining discrepancy scores with adolescent-reported parenting practices, such as a negative discrepancy score with a high adolescent-reported paternal expectation for physical activity. However, the sample size for the current study did not support this practice. Moreover, the analysis did not adjust for the number of days the adolescent lived with the father, which may influence adolescents’ perceptions of paternal activity parenting practices. Lastly, the study findings were generated from a self-selected sample who were interested in a family-focused, healthy lifestyle intervention program. Therefore, these findings may not be generalizable to all Latino early adolescents and their fathers. Nevertheless, this study focused on U.S. Latino adolescents who have a high prevalence of childhood obesity, as well as informant discrepancies between fathers and adolescents versus other studies where these discrepancies were examined primarily between mothers and adolescents [8–10]. Because Latino fathers may play an important role in childhood obesity prevention, this study provided valuable information regarding assessment of paternal involvement in promoting active lifestyles among Latino adolescents.

## Conclusion

Discrepancies in reporting activity parenting practices were evident between Latino adolescents and their fathers. Undesirable discrepancy types for certain paternal parenting practices were more likely observed in fathers without full-time employment, girls, older adolescents, and adolescents and fathers within overweight or obese BMI categories. Undesirable discrepancy types for the paternal expectation for physical activity and allowing screen time may affect adolescents’ activity levels. Future research regarding Latino fathers’ activity parenting practices could consider informant discrepancies in terms of behavioral assessments. For example, assessing adolescent-father discrepancies for activity parenting practices may be a potential strategy to identify areas of improvement regarding paternal involvement for promoting healthy, active lifestyles among Latino adolescents. Future research could also consider whether addressing these discrepancies would be an approach worth investigating in an intervention.

## Data Availability

The datasets used and/or analyzed during the current study are available from the corresponding author upon reasonable request.

## Abbreviations

BMI: Body Mass Index
MVPA: Moderate to Vigorous Physical Activity
PA: Physical Activity
ST: Screen Time
